# Medico-Physiological Researches on Animal Electricity

**Published:** 1839-10

**Authors:** 


### Art. VI.

*Recherches Medico-physiologiques sur l'Electricité Animale.* Par J. F. Coudret, m.d., &c.—*Paris,* 1837. 8vo, pp. 496. *Plates.*
*Medico-physiological Researches upon Animal Electricity.* By J. F. Coudret, m.d., &c.—*Paris,* 1837.

The subject of organic electricity, or of electricity in connexion with organized bodies, may be considered in two points of view: 1st, as it relates to the influence of electricity considered as a purely physical agent upon organized living bodies; and 2dly, as respects the electric force generated within or manifested by the organized structure itself. These two heads of enquiry are, to a certain extent at least, inseparable from each other; and the treatise before us would have been more satisfactory, as well as conceived in a more philosophical spirit, had it comprehended both branches of the subject, instead of being limited to the latter alone. The object of M. Coudret seems to have been partly to make known a new method of applying electricity as a therapeutic agent—for such in fact, allowing full force to his statements, is the correct exposition of the process adopted—and partly to develope the principles upon which, as he thinks, this remedial application rests. That he is disposed to extend these principles to a very high degree of generalization may be inferred from the motto prefixed to the *Considérations génerales,* which, with singular näiveté, he quotes from himself: "*Si le sang est le véritable aliment de la vie, le fluide électrique en est le premier moteur.*" But, however he may be disposed to felicitate himself upon the announcement of this law, the readers of the works of Abernethy and Wilson Philip will scarcely recognize M. Coudret as the founder of the electro-vital physiology. The results of previous investigations, which appear to M. Coudret as the more essential to be borne in mind, before entering upon those which are especially his own, are, "1st, that the nerves are actual organic conductors; 2dly, that electricity is to be considered as the motive principle or agent thereof; 3dly, that they present, as in the case of galvanic apparatus, two entirely different and distinct orders of currents; lastly, that one of these currents, appropriated to the functions of sensibility and intelligence, passes from the internal and external senses to the brain; the other, devoted to the nutritive and locomotive functions, is directed, on the contrary, from the brain or, if you will, from the spinal marrow to the several parts of the muscular system and of the vast sanguineo-capillary apparatus." (p. 8.) Passing over the first, third, and fourth of these propositions, the third of which we may, however, remark is unnecessarily complicated with the introduction of the loose analogy attempted to be inferred between the nervous system and a galvanic apparatus, we proceed to the examination of the second—that electricity is to be considered as the motive principle or agent of the nerves.

After stating the modern discoveries of the separate functions of the nerves of sensation and the nerves of motion, which he attributes entirely to M. Magendie, without the slightest reference to Sir Charles Bell or the other numerous cultivators of the same field of experimental research, M. Coudret brings forward his fundamental and essential facts, upon which, as he conceives, these propositions, and more especially the second of them, are established. These facts are, 1st, the well-known experiment of Galvani on the frog; 2d, the excitement of muscular motions by means of a galvanic current passed through the nerves, by which the muscles are respectively supplied; 3d, the production of muscular motion, in animals recently deprived of life, by mechanical irritation of the spinal marrow or the trunk of a motor nerve; and, lastly, the effects produced on aliment received into the stomach by division of the pneumogastric nerves, and the subsequent reestablishment of the connexion with the brain by apposition of the divided extremities of the nerves or by the interposition of a small metallic plate. This experiment has been modified by the introduction of a non-conductor between the divided ends of the nerves, in which case the food remained undigested as before, though when a metallic plate was used as the means of communication, the process of digestion went on with little or no interruption. Here again we have no mention made of Dr. Wilson Philip, to whom we owe the experiment in its original form; we must, however, do M. Coudret the justice to believe that the omission is entirely the effect of ignorance, since, in his relation of the experiment, he omits the only circumstance which renders it of any avail for the purposes for which it is referred to. The effects resulting from the renewed contact of the divided ends of the nerves only show that the nervous connexion is reestablished, and the effects of the interposition of the metallic plate merely tend to prove that the nervous influence, whatever that may be, is capable of being transmitted along the surface or through the substance of the metal, not that it is identical with or analogous to any other agent of which metals are conductors. Dr. Philip's experiment of the substitution of a galvanic current for the nervous influence and the effects thereof upon the aliment contained in the stomach are much more to the point, as they lead to the establishment of an analogy, at least, between the nervous and galvanic forces. We cannot, however, dwell further upon these points; nor need we quote the very hasty, unauthorized, and erroneous conclusions deduced from them by M. Coudret. The last of these is thus enunciated: "If in the question," he observes, "before us, as in the analysis of all possible phenomena, we must rigorously judge of the identity of the causes by the identity of the effects, we cannot henceforth doubt that the nervous agent and the electric agent are two principles perfectly identical." (p. 15.)

M. Coudret briefly alludes to the recent researches of MM. Dutrochet and Donné upon animal electricity, from which these gentlemen are disposed to conclude the existence of electric currents within the organs of the body; but he makes no mention of the many curious particulars to be found in ancient and foreign authors. The development of electricity upon the surface of certain animal bodies in consequence of friction, is a well-ascertained fact, and has probably given rise to some of the fictions of classical writers. Instances of the emission of sparks on stroking or combing the hair, and on friction of the surface of the body by the clothes, are mentioned by Cardan, Scaliger, Faber, Bartholinus, Fortunius Licetus, Gesner, Beccaria, &c. Ezekiel di Castro relates the case of a lady of Verona, resembling, in several respects, the singular one quoted from an American Journal in a recent number of this Review, (*Br. and For. Med. Rev.*, Vol. VI., p. 249.) "As often as she touched her body, even in a slight manner, with a linen cloth, it emitted sparks in great abundance, which could be perceived by every person standing near her, and were attended with considerable noise. Her maids were often deceived by this phenomenon, and believed that they had, through carelessness, dropped some coals between the sheets, as she always caused her bed to be warmed in winter, at which time the sparks were most abundant and strongest.*" These are, obviously, instances of the development of electricity without the body; and, accordingly, Saussure, who has investigated the subject of animal electricity, found that he could obtain no indications of electricity when undressed; but Hemmer, who instituted a lengthened series of experiments, both upon himself and upon several other individuals, has arrived at a different conclusion; and from these experiments it would appear that the development of electricity, although varying in intensity in different individuals, and in the same individual at different times, is yet a general property of the animal economy. For an account of these investigations we must refer to the sixth volume of the Transactions of the Electoral Society of Manheim and to the Philosophical Magazine for 1799.

To return to M. Coudret. We find him stating the following propositions as the groundwork of his subsequent investigations: first, "that every painful or inflamed part disengages a notable quantity of electricity;" and secondly, "that every means fitted to withdraw or to neutralize directly this fluid, produces the most salutary and most evident antiphlogistic and sedative effects." It appears that in January, 1833, M. Fozembas, finding himself affected for several days with severe erysipelas of the face, which he had in vain attempted to subdue by the usual means, bethought himself of having recourse to electricity. "Long entertaining the suspicion," says M. Coudret, "with us and with other physiologists of our epoch, that electricity could scarcely be otherwise than concerned in the development of similar phenomena, he resolved to make some experiments with the view of satisfying himself upon this point. Assuming that electricity is in effect one of the essential elements of vital activity, and that by its development in excess it is the principal cause of the morbid phenomena which constitute inflammation, the best means of promoting a return to the state of health should be to seek the establishment, as quickly as possible, of the natural equilibrium of the animal electricity." These means are stated to be bleeding, repose, and regulated diet, which are conceived to act as either directly or indirectly impeding the generation of electricity, and more especially the withdrawing or direct neutralization of the electricity from the inflamed part, whether by means of metallic points in communication with the ground, by topical humid applications, or by immersion of the affected part in a warm bath. All these measures are stated to have been had recourse to without benefit, with the exception of immersion in the bath, which, the face being the part affected, could not be had recourse to, and the application of the metallic points. Having contrived a suitable apparatus, which we shall presently describe, M. Fozembas proceeded to

* Castro, De igne lambente.

subject himself to a trial of this method. The metallic points of the apparatus were placed opposite to, but not in immediate contact with, the most painful and burning parts of the face.

"After some minutes' application he thought he perceived the commencement of an amelioration; but what made him soon comprehend that this opinion was not an illusion of the imagination was that, at the end of some hours, he could, for the first time, open the eyelids a little and support the impression of light, which until then he had been unable to do. Encouraged by the result of this first trial, he repeated, many times in the day, the application of the apparatus, with the precaution of causing it to act in succession upon the most irritated parts of the face. The effect was so salutary, so sensible, and so quick, that the patient and those by whom he was surrounded could scarcely credit it. Each time that the apparatus was applied for an hour to the same part, it appeared neither so red nor so tense as before, and the heat as well as the sensibility were very much lessened. At length, under the influence of this new method, the irritation and pain diminished with extreme rapidity, and not only was their rapid decrease not injurious to the patient in giving rise to some internal repercussion, but, on the contrary, from this moment the general condition continued to improve, and in a few days the restoration to health became complete." (p. 26.)

The apparatus employed in the case of M. Fozembas, to which the name of the *medical electromotor* is given, is described as follows: It consists of a box, which must be of glass or of some other isolating material, varying in size and form according to the configuration of the surface on which it is to be applied and the amount of effect intended to be produced. Deeply placed within the box is a continuous double metallic surface, of which the lower surface only is visible. This lower is set (hérissée) with a great number of very sharp points of steel. A small opening in the summit of the box affords a passage to a conducting cord several feet in length, and intended to effect a communication between the upper metallic surface and the ground. The base of the box by which it should be in connexion with the affected parts, projects a little beyond the steel points, in order that the skin may be constantly protected from the action of these points; and that this protection may be still more effective, a small network of very fine silk is extended across the framework of the base. The apparatus is attached by one or more bands of silk, intended to retain the instrument in exact apposition with the affected parts.

The mode of action of this instrument, admitting the assumption of MM. Fozembas and Coudret that a disturbance of the electric equilibrium is the principal cause of the morbid phenomena which constitutes inflammation, is sufficiently evident, although it is not equally clear that the construction of it above described is either the most philosophical or the most convenient which could have been devised. A much more important consideration here is the ascertaining of the actual existence of the alleged excess of electricity in inflammation. The method adopted by M. Coudret to prove this point is by the application of one of his electromotors upon the inflamed part, the steel points with which the under surface of the instrument is set being brought as near as possible to the skin without actual contact. An electric condenser of Volta is placed at a little distance from the patient and the conductor of the electromotor, made as short as possible and terminated by a little ball of sealing-wax, brought into contact with the superior plate of the condenser, whilst the inferior plate of the same apparatus is placed in communication with the ground. In a few minutes, interrupting the communication of the condenser with the ground and preserving at the same time the connexion of its superior plate with the electromotor, the two plates of the condenser are to be carefully separated; at the moment of this separation, the gold leaves of an electrometer which is connected with the inferior plate of the condensing apparatus will diverge, "at least," says M. Coudret, "provided no accidental cause should arise to disturb the operation and neutralize its results." The operation is one of considerable nicety and liable to numerous sources of fallacy, not the least of which is the generation of a sufficient amount of electricity to cause the divergence of the gold leaves by the friction of the plates of the condenser upon each other in the act of separation. M. Biot remarks that great attention also should be paid to avoid friction in bringing the plates of the condenser into contact, since the friction will develope a certain portion of electricity in the plates themselves which adheres strongly to them and may subsequently occasion errors in delicate experiments. Another source of fallacy is that the connexion between the inferior plate of the condenser and the ground is established by means of an assistant who places one of his fingers, "slightly moistened with saliva," in immediate contact with the lower surface of this plate. "The assistant," says M. Coudret, "destined thus to perform the functions of a conductor should be in good health, have his hands clean, and stand upon a perfect conductor of electricity, consequently by no means waxed," as is generally the case with the floors and much of the furniture in French houses; and again, "it has been demonstrated to us by a great number of observations, that if the hands of the assistant thus performing the office of a conductor are hot and dry, he may himself furnish electricity to the condenser instead of withdrawing it, and consequently vitiate the experiment." In such cases an ordinary metallic conductor is recommended to be substituted, but it must be observed that, as far as the theory is concerned, a doubt is thus thrown over the whole of M. Coudret's experiments and observations, since we have no means of ascertaining in what proportion of them these extra precautions were had recourse to. It is perhaps scarcely necessary, keeping these considerations in view, to relate any of the experiments; but as the second series narrated by M. Coudret was performed in the presence of M. Piorry, who, it may be presumed, would have pointed out any notable source of fallacy, it is but justice towards the author to mention the most striking of them, although even here we notice that the communication of the electric condenser with the ground was kept up "au moyen du doigt mouillé d'un aide." Exp. 7. Erysipelas of the face: electromotor applied upon the right cheek and placed in contact with Volta's electrometer. At the end of two minutes and a half, separation of the gold leaves to the extent of a full inch. Exp. 8. The same result from the left cheek; venesection immediately performed and the electromotor reapplied. At the expiration of two minutes and a half, separation of the gold leaves to the extent of five lines only. Exp. 10. Electromotor applied to the epigastrium of M. Grossetête, a student of medicine enjoying good health, but having the epigastric region very hot. The gold leaves of the electrometer separated to the extent of four lines.

Exp. 11. Performed on M. Belonius, also a medical pupil, possessed, as he stated, of strong magnetic powers (d'une force magnétique prononcée.) Electromotor applied for two minutes on the right hand, which was excessively dry but of a temperature but slightly elevated. Separation of the leaves to nearly four lines. On the epigastrium of the same subject the separation was nearly the same as on the hand, though the temperature seemed rather less, at least to the touch.

From a careful examination of the experiments above referred to, as well as others related by M. Coudret, it does not appear to us, setting aside the obvious sources of fallacy to which we have already alluded, that the effects upon the electrometer are necessarily to be attributed to the generation or accumulation of electricity in inflamed parts; or, indeed, that they were at all connected with a purely electric condition, even of the mere surface of the body. The recent researches into thermo-electricity would seem to afford sufficient grounds for attributing the electric effects observed to the induction of electricity by heat alone. M. Becquerel conceives that "whenever a particle of a metal is heated, part of the neutral electric fluid which is attached to it is decomposed, the vitreous fluid being retained, and the resinous driven off and passing into the adjoining particles. In proportion as the heat extends by communication from particle to particle, similar effects take place in each of those that are acquiring heat, and the contrary in those that are losing it. Thus the first effect is only to produce an oscillatory movement of the electric fluid between the adjacent particles; but if the source of heat be permanent, the retrograde movements are prevented, and a continued current takes place."* In the second volume of the Annales de Chimie et du Physique is a paper by M. Dessaignes, in which several curious experiments are related, also tending to prove the development of electricity in metallic bodies, merely by change of temperature, some of which are remarkably analogous to the recent investigations connected with thermo-electricity. In confirmation of the view here advanced, we may state that a similar idea seems to have suggested itself to M. Piorry; for we find M. Coudret "wishing to show to M. Piorry, first, that the electromotor employed to prove the presence of animal electricity, did not of itself develope electricity; secondly, that the action of the temperature of the human body upon this apparatus could not be considered as the cause of the phenomenon:" and then proceeding to perform certain experiments upon other heated bodies, which, however, do not appear to us by any means calculated to meet the objection.

Assuming the explanation which we have here given to be correct, the electromotor of M. Coudret is in fact a thermo-electric apparatus, and being placed in connexion with the heated surface of the human body, induction of electricity by heat ensues. The electricity thus developed is conducted to the condenser, and, according to the preceding statement of the views of M. Becquerel, should be of the resinous or negative kind. Now this is precisely what M. Coudret found to be the case: "everything,' he says, "leads us to consider as certain that the electric fluid condensed in our inflamed tissues is always negative;" or, in other words, that the electricity manifested by the electrometer in connexion with an electro-

* Report on Thermo-electricity. By Professor Cumming. Proceedings of British Association for 1832.

motor placed over inflamed parts is in all cases negative. According to these views then, the electricity is generated in the apparatus itself by means of heat, the negative fluid being conveyed off towards the ground, and, if any interchange takes place between the surface of the body and the instrument, the vitreous or positive fluid being communicated to the part over which the instrument is applied, affording thus, as we stated at the commencement of this article, a new method of applying electricity, as a therapeutic agent.

One other observation may be made respecting what may be termed the physiological effects of electricity before turning our attention to the practical part of the subject. "In a vast number of cases," says M. Coudret, "but especially when the instrument is applied to the head, a general moisture arises, and at the same time a drowsiness or sleep more or less profound; in some circumstances this moisture does not extend much beyond the small space occupied by the instrument and is not accompanied by sleep." How far the drowsiness or sleep here alluded to is the effect of the application of the remedy, or whether it is to be attributed to a strong impulse on the imagination of the patient is perhaps questionable, but its analogy with the coma developed in the course of the manipulations of animal magnetism cannot fail to suggest itself. The moisture or perspiration, whether general or local, is no more than what has been noticed in the communication of electricity by other modes. The Abbé Nollet found, by correct statical experiments, that an electric current communicated to a living animal for a considerable time greatly increased the amount of perspiration; and Van Marum, Read, and others have noticed the same. From the consideration of these and other facts, we are led to conclude that the communication of electricity in the mode recommended by M. Coudret, tends to increase the capillary circulation, and therefore that in accordance with the views of the intimate nature of inflammation entertained by Dr. Hastings* and others, it enables the capillary vessels to unload themselves of their contents, thus relieving that state of inaction and consequent congestion, which, according to these authorities, constitutes the proximate cause or rather the very essence of inflammation. Upon these principles we shall have no difficulty in understanding and accounting for the therapeutical effects of M. Coudret's method of applying this remedial agent.

A considerable portion of the treatise before us is occupied with the narration of cases of ophthalmia, erysipelas, cephalalgia, hemierania, and other cerebral affections, catamenial disorders, rheumatic, neuralgic, and other painful affections, &c., in the greater number of which the electromotor is extolled as a heroic remedy. It appears, however, to occupy in these cases precisely the place which stimulant applications to the eye do in certain states of ophthalmia, as the subjoined note of the author on the case of M. Fozembas sufficiently testifies. The rapidity of the cure in this case was owing to the patient having been prepared for the remedy by the debilitating means and regimen previously employed. "*In every case where sanguineous plethora exists, it must in the first instance be removed, either by bleeding or starvation* (*diète sévère*); as soon as the irritation becomes merely local, the electromotor produces

* Treatise on Inflammation of the Mucous Membrane of the Lungs.

the most remarkable effects." We had marked one or two of the cases for quotation, but as they are mostly similar in their general features to that of M. Fozembas, already noticed, we have the less hesitation in passing them over. The concluding pages, amounting to about one fifth of the whole work, are occupied with hypothetical considerations founded on the electro-medical system. They relate to the classification of medicines upon electro-medical principles, and embrace certain views in connexion with the same upon sensibility and sensation, on the nature and origin of cholera, on temperaments, aliment and drinks, atmospheric air, clothing, &c.

---

